# Gasparyan Method of Total Autologous Reconstruction of the Aortic Valve

**DOI:** 10.21470/1678-9741-2020-0197

**Published:** 2020

**Authors:** Vahe Gasparyan

**Affiliations:** 1 Erebouni Medical Center, Yerevan, Armenia.

**Keywords:** Aortic, Valve Insufficiency, Aortic Valve, Glutaral, Polypropylenes, Echocardiography, Transesophageal, Pericardium, Aorta, Sutures, Running

## Abstract

In this case report, I describe a new technique for total reconstruction of the aortic valve with autologous pericardium. The parameters of the cusps were calculated using very simple formulas after measurement of the aortic root intercommissural distances. Glutaraldehyde-treated pericardium was trimmed along the marked line, leaving 2 mm of tissue along the fibrous annulus attachment margin for the suture and small wings on both commissural margins to secure the commissural coaptation between right and noncoronary cusps. The annular margin of each pericardial cusp was sutured to the corresponding fibrous annulus with running 4/0 polypropylene suture. The commissures of pericardial patch and the commissural coaptation between right and noncoronary cusps were secured with mattress 4/0 polypropylene sutures. The coaptation of the three cusps was checked with negative pressure on the left ventricular vent before closure of the aortotomy. Intraoperative transesophageal echocardiogram revealed a peak pressure gradient of 10 mmHg and trivial aortic regurgitation.

**Table:** 

Abbreviations, acronyms & symbols	
**lL**	**= Intercommissural distance of left coronary cusps**
**l_mean_**	**= Mean intercommissural distance**
**lN**	**= Intercommissural distance of noncoronary cusps**
**lR**	**= Intercommissural distance of right coronary cusps**

## INTRODUCTION

Total autologous reconstruction of the aortic valve is currently gaining more popularity. I describe a new technique of this operation without any templates, which makes this operation cheaper, easier and more reproducible.

## TECHNIQUE OF PROCEDURE

**Fig. 1:**
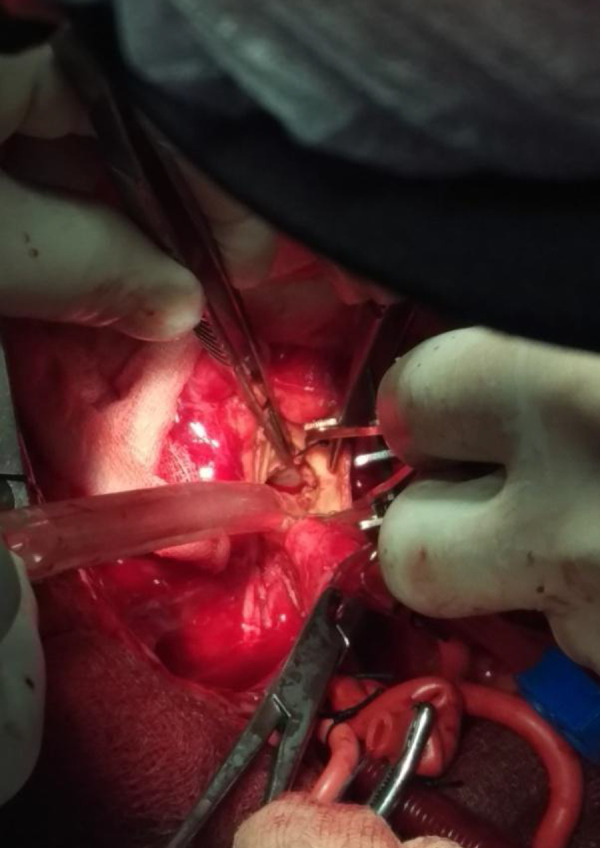
Sizing of the intercommissural distances of right, left and noncoronary cusps.

New technique of total autologous aortic valve reconstruction was used in a 39-year-old patient who presented with septic endocarditis and aortic stenosis (peak pressure gradient through the aortic valve of 80 mmHg) complicated with small aortic root (17 mm) and fibrous annulus abscess. He was treated with antibiotics for 6 weeks before surgery. The patient underwent this operation after obtaining written informed consent. An 8 × 10 cm piece of pericardium was harvested after the usual median sternotomy. The harvested pericardium was treated with 0,625% glutaraldehyde solution for 10 minutes. Cardiopulmonary bypass was established with aortic and right atrial cannulation. The heart was arrested in diastole by retrograde cold blood cardioplegia. The arrest was maintained with retrograde cold blood cardioplegia every 20 minutes. A transverse aortotomy was performed 1 cm distal from the sinotubular level. After resection of the diseased aortic cusps, the sizes of the intercommissural distances of right (lR), left (lL) and noncoronary (lN) cusps were taken with calipers (Figure 1) and compared to a ruler (to the nearest 0,5 mm). The mean intercommissural distance (l_mean_) was calculated as following:

$$I_{\mathrm{mean}}=\left(\mathrm{IR}+\mathrm{IL}+\mathrm{IN}\right)/3$$

The aortic valve parameters were calculated (Table 1) using my formulas, published in 2000^6^:

$$\begin{array}{c} \mathrm{LR}=1.2\times \mathrm{lR}\\ \mathrm{LL}=1.2\times \mathrm{lL}\\ \mathrm{LN}=1.2\times \mathrm{lN}\\ H=0.866\times l_{\mathrm{mean}}\\ R=0.6\times l_{\mathrm{mean}}\\ K=0.266\times l_{\mathrm{mean}}, \end{array}$$

**Table 1:** Measured and calculated parameters of aortic root and valve cusps.

Measured parameters (mm)			Calculated parameters (mm)						
lR	lL	lN	l_mean_	LR	LL	LN	H	R	K
14	13	13	13.3	17	16	16	12	8	4

where LR, LL and LN are the lengths of coapting edge of right, left and noncoronary cusps, respectively; H is the cusps height; R is the annular margin line radius and K is the commissural height. The parameters of the new valve were marked on the pericardium leaving a distance of about 4 mm between the cusps (Figure 2) as the pericardial commissures. The glutaraldehyde-treated pericardium was trimmed along the marked line (dashed line in Figure 2) leaving about 2 mm of tissue along the fibrous annulus attachment margin for the suture and small wings on both commissural margins to secure the commissural coaptation between right and noncoronary cusps. The coapting margins of the pericardial leaflets were trimmed not as a straight line, but slightly as a pyramid-shape line (Figure 2) to raise up the contact point of the new pericardial cusps for better coaptation. The annular margin of each pericardial cusp was sutured to the corresponding fibrous annulus with running 4/0 polypropylene suture (Prolene, Ethicon, Inc., Somerville, NJ) as described in my previous publication^[1]^. The commissures of pericardial patch and the commissural coaptation between right and noncoronary cusps were secured with mattress 4/0 polypropylene sutures as described in my previous publication^[1]^. The smooth (inner) surface of pericardium was placed on the left ventricular side. The coaptation of the three cusps was checked with negative pressure on the left ventricular vent before closure of the aortotomy (Figure 3). Intraoperative transesophageal echocardiography revealed peak pressure gradient of 10 mmHg and trivial aortic regurgitation. The patient did not take anticoagulants postoperatively. Follow-up transthoracic echocardiography performed 1 and 3 months after surgery revealed a peak pressure gradient of 12 mmHg and grade I aortic regurgitation.

**Fig. 2:**
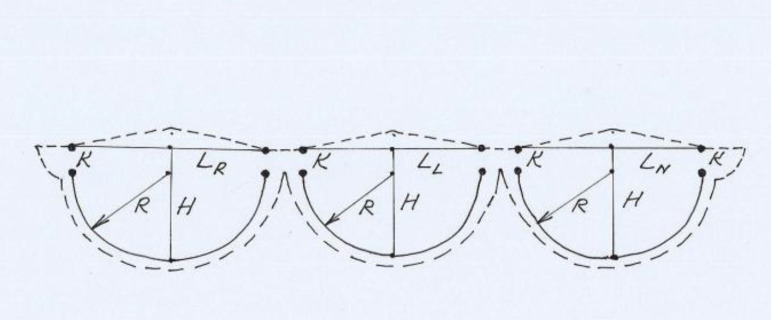
Marking of the calculated parameters of the aortic valve in the pericardium (LR, LL and LN are the lengths of coapting edge of right, left and noncoronary cusps, respectively; H is the cusps height; R is the annular margin line radius and K is the commissural height).

**Fig. 3:**
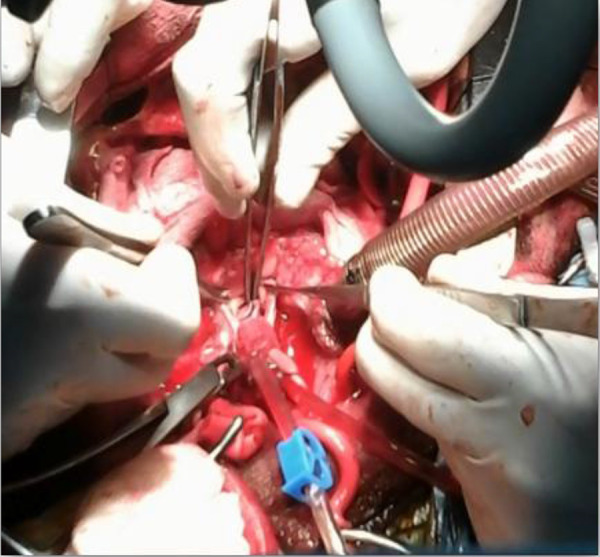
Final view of the new pericardial valve sutured to the fibrous annulus of the root. Good coaptation of the pericardial leaflets is seen.

## DISCUSSION

Total reconstruction of aortic valve with glutaraldehyde-treated autologous pericardium is currently gaining more popularity. This is a stentless, autologous valve - a very good alternative for the prosthetic valves and Ross operation. It was firstly reported by Dr. Duran back in 1995^[2]^. Special molds of different sizes depending on the aortic root diameter were used to tailor the pericardial patch. Dr. Ozaki has recently reported his experience with this operation using self-developed sizing apparatus and templates for pericardial trimming^[3,4]^. They reported very good results in the medium and long term^[3-5]^. All authors still used molds or templates for pericardial patch trimming. The described method is cheaper, easier and more reproducible, since no templates are necessary, and all parameters of the cusps can be calculated using very simple formulas^[6]^. Tailoring of the pericardial valve is not difficult and took only about 10 minutes. The overall cross-clamp time was 76 minutes, which is quite acceptable.

## CONCLUSION

This is an original method of total autologous reconstruction of the aortic valve. It is cheaper and easier than other similar techniques described in the literature, since no molds or templates are necessary and all parameters of the new valve cusps can be calculated using very simple formulas after intraoperative measurement of the root intercommissural distances. I believe that the described technique will make this operation more reproducible.

**Table:** 

Author's roles & responsibilities	
VG	Substantial contributions to the conception or design of the work; or the acquisition, analysis, or interpretation of data for the work; drafting the work or revising it critically for important intellectual content; final approval of the version to be published
